# Raman Spectroscopy Measurements Support Disorder-Driven Capacitance in Nanoporous Carbons

**DOI:** 10.1021/jacs.4c10214

**Published:** 2024-11-01

**Authors:** Xinyu Liu, Jaehoon Choi, Zhen Xu, Clare P. Grey, Simon Fleischmann, Alexander C. Forse

**Affiliations:** † Yusuf Hamied Department of Chemistry, University of Cambridge, Cambridge CB2 1EW, U.K.; ‡ Helmholtz Institute Ulm (HIU), 89081 Ulm, Germany; § Karlsruhe Institute of Technology (KIT), 76021 Karlsruhe, Germany

## Abstract

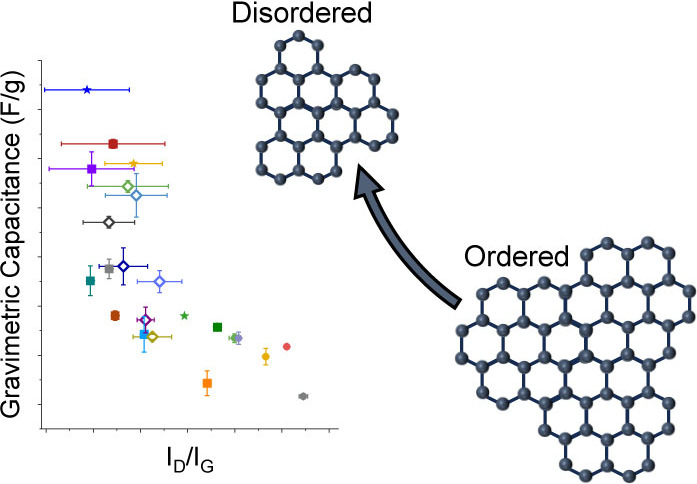

Our recent study of 20 nanoporous activated carbons showed that a more disordered local carbon structure leads to enhanced capacitive performance in electrochemical double layer capacitors. Specifically, NMR spectroscopy measurements and simulations of electrolyte-soaked carbons evidenced that nanoporous carbons with smaller graphene-like domains have larger capacitances. In this study, we use Raman spectroscopy, a common probe of local structural disorder in nanoporous carbons, to test the disorder-driven capacitance theory. It is found that nanoporous carbons with broader D bands and smaller I_D_/I_G_ intensity ratios exhibit higher capacitance. Most notably, the I_D_/I_G_ intensity ratio probes the in-plane sizes of graphene-like domains and supports the findings from NMR that smaller graphene-like domains correlate with larger capacitances. This study supports our finding that disorder is a key metric for high capacitance in nanoporous carbons and shows that Raman spectroscopy is a powerful technique that allows rapid screening to identify nanoporous carbons with superior performance in supercapacitors.

Electrochemical double layer capacitors (EDLCs) are promising energy storage devices with high power densities and long cycle lives.^1,2^ Nanoporous carbons, especially activated carbons, are the cheapest and most commonly used electrode materials in commercial EDLCs.^2^ Such carbons consist of disordered graphene-like fragments containing defects. These fragments form the pore walls of porous three-dimensional structures with a distribution of pores sizes.^3^ The impact of a range of structural factors that control the capacitance of nanoporous carbons have been explored including the material’s surface area,^4^ pore size^5−10^ and surface chemistry,^11−13^ although accepted design principles for carbons with improved capacitance have been lacking.

Our recent study explored the use of NMR spectroscopy of electrolyte-soaked carbons to determine which structural factors correlate best with capacitance. The study of 20 predominantly microporous activated carbons (i.e., carbons where most pores are below 2 nm in diameter) showed that the degree of disorder in the graphene-like sheets correlates with their capacitance.^14^ Specifically, measurements of the in-plane sizes of the graphene-like domains were carried out with a combination of solid-state nuclear magnetic resonance (NMR) spectroscopy measurements and a lattice-simulation model.^14−16^ This NMR approach probes the in-plane domain sizes of the different carbons via the nuclear independent chemical shifts (NICS) of adsorbed electrolyte ions. The lattice-simulations decouple the effects of the domain sizes and the pore size on the chemical shifts for adsorbed species, allowing extraction of graphene-like domain sizes.^14−16^ Crucially, we found that the carbons with smaller graphene-like domains have higher capacitances in symmetric EDLCs when combined with a conventional organic electrolyte. Given the relatively high cost of NMR equipment and the requirement for spectral simulations to extract domain sizes, we sought a more accessible technique to explore and test disorder-driven capacitance theory.

Raman spectroscopy is a well-established and powerful technique to study structural disorder in carbonaceous materials.^17−19^ Two major features are commonly observed in the Raman spectra of disordered carbons. The D band (between 1330 and 1350 cm^–1^) is assigned to the A_1g_ breathing mode of the six membered carbon rings in a graphene sheet, which is only allowed when disorder is present, breaking the 6-fold symmetry of the graphene sheet. The G band (between 1580 and 1590 cm^–1^) is attributed to the E_2g_ stretching mode of the sp^2^ bonds, and is allowed for both ordered and disordered carbons.^20−22^ Robertson and Ferrari have proposed a 3-stage model to interpret the Raman spectra of carbons with varying degrees of structural order, ranging from ordered graphite to amorphous (tetrahedrally coordinated) carbon with high sp^3^ content.^22^ Highly disordered nanoporous carbons with in-plane crystalline correlation lengths (L_a_) less than 2 nm, as indicated in X-ray pair distribution function patterns,^14,19^ fall into stage 2 of this model, and the intensity of the D-band is now proportional to the probability of finding a six-membered (aromatic) ring in the graphene-like cluster or sheet—and is thus proportional to the cluster area: a stronger D band intensity indicates a larger graphene-like domain size in the carbon structure. As a result, the I_D_/I_G_ intensity ratio is proportional to the square of the in-plane correlation length, L_a_. In addition, a distribution in the domain and ring sizes (i.e., six membered vs five and seven etc. membered rings) of the graphene-like fragments gives rise to the broadening of the D band.^22^ The full width at half-maximum (FWHM) of the D-band, therefore, allows for another qualitative comparison of structural disorder distributions among different carbons. Additionally, a broad feature appears at 2300 to 3200 cm^–1^, that is attributed to the modulated 2D, D+D′ and 2D′ bands.^22,23^ We note that the poorly crystalline carbons studied here give rise to extremely broad Bragg reflections so that while L_a_ can be estimated from the total scattering, attempts to extract it from the Bragg reflections are not reliable.^19,24^ Here, our comparative study of disorder measurements from Raman and NMR spectroscopy yields new insight into the structures of amorphous nanoporous carbons and provides new evidence for disorder-driven capacitance.

Figure 1 shows the Raman spectra of 10 commercial nanoporous carbons from different producers (see Figure S1 for comparison with NMR spectra), and three titanium carbide derived carbons (TiC-CDC) from previous literature.^5,19^ The spectra are sorted according to the capacitance of the carbons in symmetric EDLCs with a standard organic electrolyte (Table S1). All Raman spectra exhibit the expected two major components centered around 1340 and 1590 cm^–1^, corresponding to the D and G bands, respectively. The D bands of the best performing carbons, TiC-CDC-600, ACS-PC, TiC-CDC-800, and SC-1800, are evidently broader, indicating a wider distribution of different domain sizes as compared with the other studied carbons.^22^ This provides initial evidence that nanoporous carbons with broader distributions of domain sizes exhibit an enhanced performance in EDLCs. In addition, the (2D, D+D′ and 2D′) peaks at 2300 to 3300 cm^–1^ are extremely broad and overlapping for TiC-CDC-600, ACS-PC, TiC-CDC-800, and SC-1800, compared with the other lower capacitance carbons, which show more well-defined second-order peaks (Figure S2).

**Figure 1:**
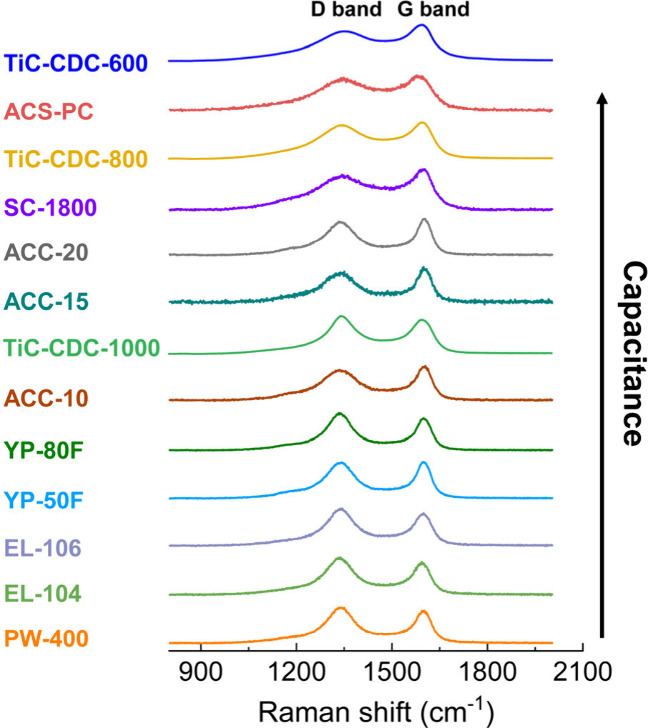
Raman spectra (532 nm) of ten commercial nanoporous carbons, with literature spectra also shown for three TiC-CDCs (532 nm)^5,19^ which are reproduced with permission from ref (19), copyright [2015] American Chemical Society. Raman spectra are plotted in order of their increasing capacitance. Raman spectra were also acquired at multiple spots and using various spot sizes (Figures S3–S5) in consideration of the potential inhomogeneity of the carbons. Gravimetric capacitances of commercial nanoporous carbons were measured in two-electrode symmetric coin cells at 0.05 A/g in 1 M NEt_4_BF_4_ (acetonitrile, ACN). Capacitances of TiC-CDCs were extracted from previous literature,^5^ and were measured in two-electrode cell at 5 mA/cm^2^ in 1.5 M NEt_4_BF_4_ (ACN) (Table S1), with that data reproduced with permission from ref (5), copyright [2006] The American Association for the Advancement of Science.

For further interpretation of the Raman spectra, peak deconvolution of the D and G band region was conducted with a four-peak model which takes two “peak shoulders” into consideration.^20,24−26^ These two additional small peaks centered at around 1500 cm^–1^ (D′′ band) and 1200 cm^–1^ (I band) arise from the activation of forbidden vibration modes due to structural disorder, consistent with previous studies.^24,27−29^ It is noted that a simple two-peak model was not able to provide good fits across the full series of carbons (Figure S6).

With a data set of 23 nanoporous carbons including 10 commercial activated carbons, their thermally annealed counterparts at varying temperatures (Figure S7),^14^ and three TiC-CDCs from previous studies,^5,19^Figure 2a and b show correlations of the fitted Raman parameters with capacitances obtained from symmetric EDLCs with organic electrolyte (1 M NEt_4_BF_4_ in acetonitrile). First, carbons with larger D band FWHMs tend to have higher capacitances (Figure 2a). This supports the idea introduced above that carbons with broader distributions of ring sizes and graphene-like domain sizes exhibit enhanced capacitances. Second, it is observed that carbons with smaller I_D_/I_G_ values have higher capacitances (Figure 2b), supporting the previous findings from NMR spectroscopy that carbons with smaller graphene-like domain sizes have higher capacitances (Figure 2b).^14,21,22,24,30^ While our graphs in Figure 2 show capacitances for slow charging (0.05 A/g), similar correlations were observed at a faster charging rate of 1 A/g (Figure S11), although one outlier appears for a carbon with a very small pore size.^14^ Strikingly, the plots of (i) capacitance vs I_D_/I_G_ (Figure 2b) and (ii) capacitance vs the NMR-derived ordered area (Figure 2c)^14^ show very similar correlations. This can be rationalized since in Stage 2 of Robertson and Ferrari’s model the I_D_/I_G_ value is proportional to the area of the ordered graphene-like domains (the square of L_a_),^22^ i.e., both correlations show that capacitance increases for porous carbons with smaller graphene-like domains (Figure 2b, c).

**Figure 2:**
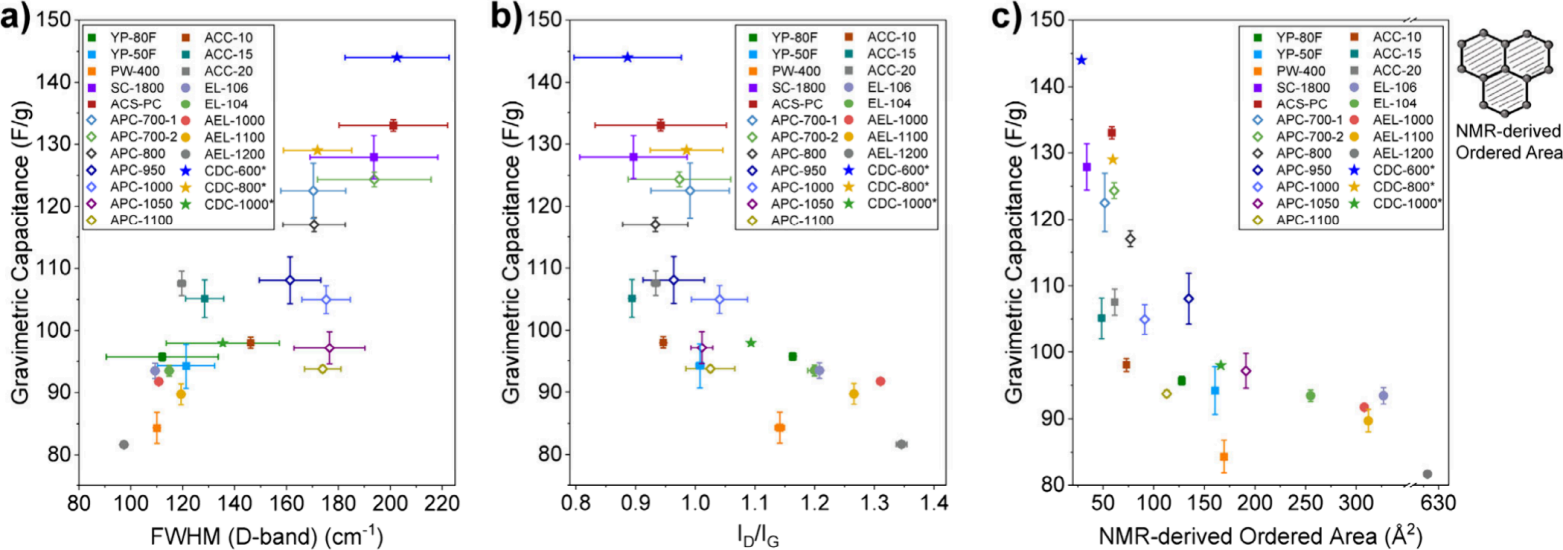
(a) Relationship between gravimetric capacitance and D band FWHM of the studied carbons. (b) Relationship between gravimetric capacitance and the I_D_/I_G_ intensity ratio (i.e., peak height intensity ratio) of the studied carbons. (c) Relationship between gravimetric capacitance and ordered area derived from NMR simulations of the studied carbons, with a schematic illustration of the ordered area on the right. Gravimetric capacitances of commercial nanoporous carbons and their thermally annealed counterparts were measured in two-electrode symmetric coin cells at 0.05 A/g in 1 M NEt_4_BF_4_ (acetonitrile, ACN) (Figure S8). Capacitances of TiC-CDCs were extracted from previous literature,^5^ measured in two-electrode cell at 5 mA/cm^2^ in 1.5 M NEt_4_BF_4_ (ACN), with that data reproduced with permission from ref (5), copyright [2006] The American Association for the Advancement of Science. The error bars of the capacitance represent the standard deviation of at least two repetitive cells per carbon. The error bars of the Raman parameters demonstrate the standard deviation of fits from three independent researchers (see experimental section and Figures S9 and S10 for results from other fitting methods). See Figure S11 for the relationship between gravimetric capacitance at higher current density (1 A/g) and D band FWHM, as well as the I_D_/I_G_ intensity ratio. At a higher current density, an outlier appears, suggesting that other structural factors beyond the degree of order influence the kinetic performance, e.g. pore size. See our previous study for the pore size distributions of the studied carbons.^14^

To further compare the insights from the two techniques, we plotted the ordered areas from NMR simulations against the I_D_/I_G_ intensity ratios from Raman spectroscopy (Figure 3). Nanoporous carbons with smaller ordered areas from NMR simulations also show smaller I_D_/I_G_ values, with a linear correlation between the two parameters (Figure 3). This supports the idea above that the two techniques provide two different measurements of the areas of the graphene-like domains and supports the use of the I_D_/I_G_ intensity ratio as an appropriate probe of the graphene-like domain size. Note that we also show Raman fitting results using A_D_/A_G_ integral ratios in Figure S10 (i.e., ratios obtained by using the peak areas), with the A_D_/A_G_ ratios providing a weaker correlation with capacitance, presumably because the D-band FWHM correlates with disorder while the peak height correlates with order.

**Figure 3:**
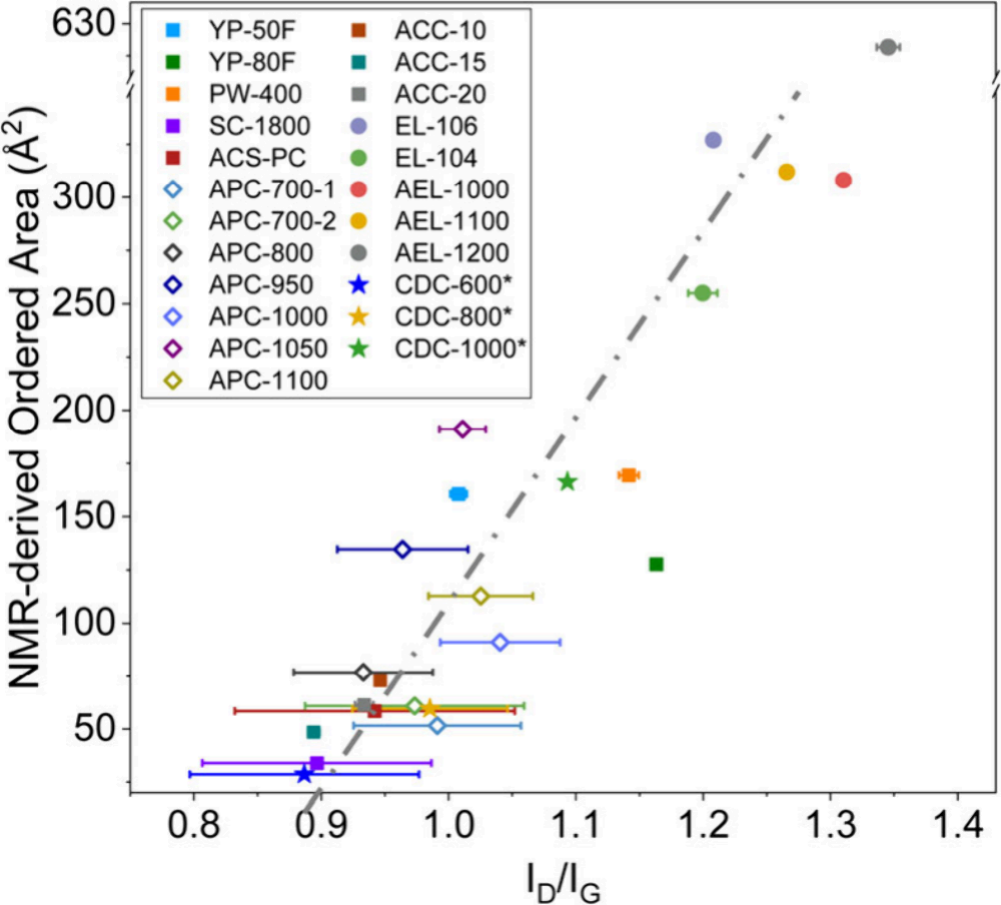
Correlation between the ordered area from NMR simulations and I_D_/I_G_ intensity ratio of the studied carbons. The error bars of the Raman parameters demonstrate the standard deviation of fits from three independent researchers (see experimental section) and reflect the difficulty associated with deconvoluting overlapping peaks. The error bars are larger for the more disordered carbons due to increased peak overlap.

There is an important distinction between the two different spectroscopic probes (Raman and NMR) of the areas of the graphene-like domains, which may be manifested in future measurements of a wider range of carbon materials. Specifically, the NMR spectroscopy approach measures structural disorder as experienced by the electrolyte ions adsorbed in the carbon pores (achieved through measurement and simulation of the ring current shifts for the adsorbed electrolyte ions).^14,15^ In contrast, the Raman spectra measure the carbon disorder irrespective of any electrolyte sorption and provide an overall measurement of disorder throughout the carbon, regardless of whether electrolyte ions can actually access the various carbon surfaces. Therefore, the NMR approach provides more targeted information, providing both the in-plane domain sizes, as experienced by adsorbed ions, and the in-pore ion adsorption capacities via integration of the resonances from in-pore ions.^14^

For the series of carbons studied here, all the materials are highly porous with a BET surface area larger than 1000 m^2^/g, and the electrolyte ions are able to access the material porosity in all cases.^14^ In this scenario, the ordered areas measured by NMR and the I_D_/I_G_ ratio are correlated with each other (Figure 3), and both are inversely correlated with capacitance (Figure 2b, c). However, we should also consider the hypothetical scenario of a porous carbon with very limited porosity accessible to the electrolyte ions—a case where low capacitance would be expected. In this scenario, the ordered area probed by NMR spectroscopy may no longer be correlated with the I_D_/I_G_ ratio from Raman. While the Raman spectra would still measure carbon disorder, they would give no indication of whether electrolyte can access the pores or not. In contrast, the NMR spectra would reveal a very low uptake of electrolyte ions in the carbon pores (through a low in-pore peak integral), and would lead to the expectation of low capacitance.

Finally, it is noted that many other factors may affect capacitance beyond the carbon “back-bone” or “framework”. The disorder versus capacitance analysis has been performed here with capacitance values measured in an organic electrolyte. However, we and others have noted that functional groups beyond the C–C bonds that form the amorphous carbons backbone may alter the overall charge of the carbon (as often quantified via a point of zero charge (PZC)).^31,32^ Particularly in aqueous electrolytes, where many of these functional groups (*e.g*., phenols and chromenes) can be protonated or deprotonated depending on pH, the role of these functional groups may also need to be considered. However, both NMR and Raman provide well established methods for identifying and quantifying these species.

In conclusion, Raman spectra of a large series of microporous carbons provide new evidence for disorder-driven capacitance. Nanoporous carbons with smaller I_D_/I_G_ ratios and therefore smaller graphene-like domain sizes exhibited higher capacitances. This closely mirrors our previous findings where smaller graphene-like domain sizes measured with NMR spectroscopy and simulations also correlated with higher capacitances. Additionally, carbons with broader D bands and thus a wider range of different carbon coherence lengths also tended to have higher capacitance. This study further shows that Raman spectroscopy is a powerful technique for the fast screening of nanoporous carbons for enhanced performance in EDLCs; this can be coupled with the NMR approach, which has the key benefit of providing information on the ion accessible porosity alongside disorder metrics.

## Data Availability

All data are available in the main text or the Supporting Information. All raw experimental data files are available in the Cambridge Research Repository, Apollo. DOI: 10.17863/CAM.109009.

## Supporting Information Available

Experimental details, Raman spectra, NMR spectra, deconvolution of Raman spectra, Relationship between gravimetric capacitance and parameters derived from Raman spectra using other fitting methods, and a table of the gravimetric capacitance of all studied carbons. (PDF)
