# Supplementary material for: Raman Spectroscopy Measurements Support Disorder-Driven Capacitance in Nanoporous Carbons
Source: J Am Chem Soc. 2024 Nov 1;146(45):30748–52. doi: 10.1021/jacs.4c10214 (PMC11565708; doi:10.1021/jacs.4c10214)
Supplement: Supplementary file 1 — ja4c10214_si_001.pdf [file ja4c10214_si_001.pdf]

Supporting Information: Raman Spectroscopy Measurements Support Disorder-driven Capacitance in Nanoporous Carbons

Xinyu Liu,[1] Jaehoon Choi,[2,3] Zhen Xu,[1] Clare P. Grey,[1*] Simon Fleischmann,[2,3*] Alexander C. Forse[1*]

1 Yusuf Hamied Department of Chemistry, University of Cambridge, Cambridge CB2 1EW, U. K.

2 Helmholtz Institute Ulm (HIU), 89081 Ulm, Germany

3 Karlsruhe Institute of Technology (KIT), 76021 Karlsruhe, Germany

Experimental section

Materials:

YP50F and YP80F activated carbon powders were obtained from Kuraray (Japan), PW-400 activated carbon, SC-1800 activated carbon and ACS porous carbon powders were obtained from Carbon Activated Corp. (America), Puragen Activated Carbon, and Advanced Chemicals Supplier Material, respectively. Activated carbon cloths ACC-5092-10, ACC-5092-15 and ACC-5092-20 were purchased from Kynol. PowerSorb EL-106 and PowerSorb EL-104 activated carbon powders were obtained from Jacobi (Jacobi Carbons Group). The thermal annealing of ACS-PC and EL-104 were conducted using a Carbolite tube furnace in a horizontal ceramic tube under a set temperature with argon gas flow at 60 cm^3/min. See our previous study for detailed description.[1]

Raman Spectroscopy:

Raman spectroscopy was carried out with a Renishaw inVia confocal Raman microscope equipped with a 532 nm laser. The spectra were recorded with an acquisition time of 10 s and two accumulations using a laser output power of 2.5 mW. The maximum 532 nm laser power is 500 mW and the spectra were collected using 0.5 % power. The averaged Raman spectra were averaged over three to four spectra for each sample.

For four-peak analysis, all Raman spectra were normalized by maximum intensity, baseline-subtracted and deconvoluted using a four-peak model with Lorentzian function. Fit parameters included peak positions, full widths at half maximum (FWHM), and peak areas. Each spectrum was deconvoluted for three times by three different researchers from HIU and Cambridge. For each fitting, different initial peak positions were applied, then varied freely to achieve the optimal fitting. The results in Figure 2 represent the average of the three fits, with the error bars showing the standard deviation among the different fits. For four-peak analysis with constraints, the position of the left hand “shoulder” peak was kept consistent between 1180 to 1200 cm^{-1}, and the position of the right hand “shoulder was maintained at 1500 to 1530 cm^{-1} for all spectra. The full widths at half maximum (FWHM) and peak areas were varied freely to achieve the optimal fitting.

For two-peak analysis, all Raman spectra were normalized by maximum intensity, baseline-subtracted and deconvoluted using a two-peak model with Lorentzian function. Fit parameters included peak positions, full widths at half maximum (FWHM), and peak areas. For each fitting, different initial peak positions were applied, then varied freely to achieve the optimal fitting.

NMR spectroscopy:

All NMR measurements and NMR-derived ordered areas are from our previous work.[1] Briefly, NMR spectroscopy experiments were carried out carbon samples soaked with 1 M NEt_4BF_4 in acetonitrile electrolyte. Measurements were performed with a Bruker Avance Neo spectrometer in a Bruker 2.5 mm HX double resonance probe. Measurements were carried out at a magnetic field strength of 9.4 T, corresponding to a ^{1}H Larmor frequency of 400.1 MHz. All spectra were acquired with a 90°pulse-acquire sequence at a sample spinning speed of 5 kHz. ^{19}F NMR spectra were referenced relative to neat hexafluorobenzene (C_6F_6) at −164.9 ppm. The NMR simulations were carried out using previously developed lattice model (see our previous work).[1-3]

Electrochemical Measurements:

All capacitance measurements are from our previous work.[1] Briefly, all electrochemical measurements were conducted in a two-electrode coin cell configuration with a Biologic BCS-805 battery cycler. The reported capacitance was calculated from constant charge-discharge measurements at 0.05 A/g with a voltage window of 0-2.5 V, with at least 2 repeat cells for each material. To check the if the cell pre-cycling process was sufficient, the constant current charge-discharge measurements at 0.05 A/g were conducted again after all measurements for all samples (including the annealed samples) to check if the capacitance values changed due to pore wetting effects. No increase in capacitance was observed, suggesting sufficient pre-activation (Figure. S8). See our previous work for more detail.[1]

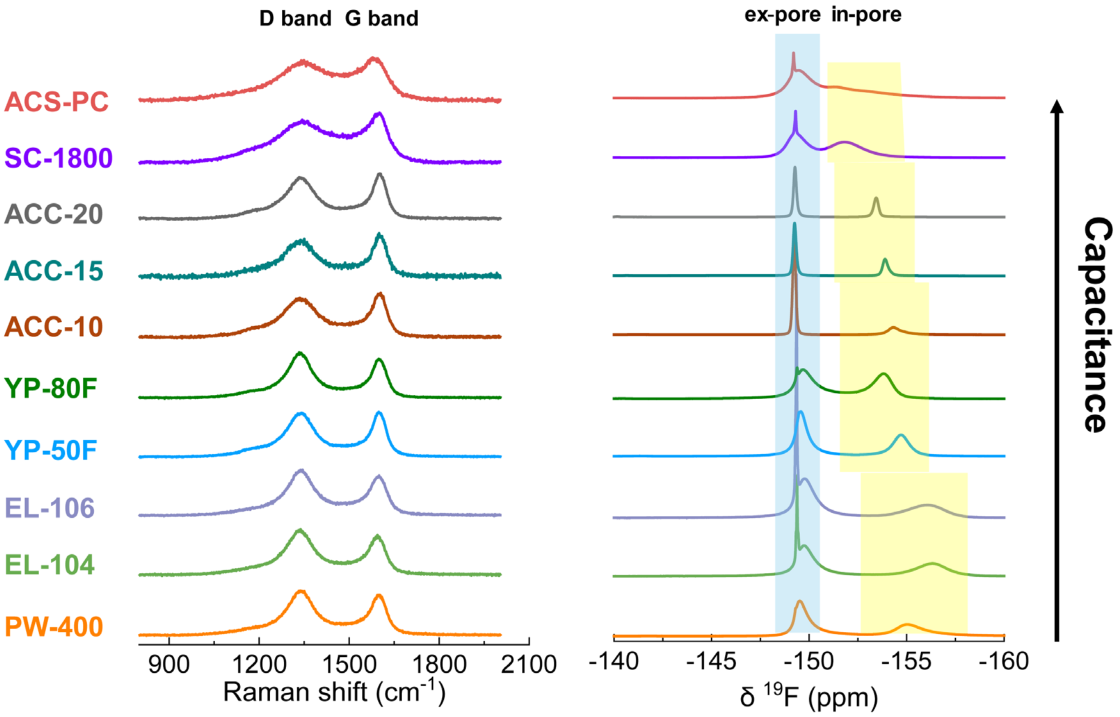

Figure S1. Comparison between Raman spectra (532 nm) (left) of commercial nanoporous carbons and ^{19}F MAS NMR spectra (9.4 T, 5 kHz MAS) (right) of commercial nanoporous carbons saturated with 1 M NEt_4BF_4 (ACN). For the carbons with the highest capacitances, broad Raman features are observed, and smaller in-pore (shaded in yellow) –neat electrolyte (-149.3 ppm) chemical shift differences are seen for in-pore anions. See our previous work for more information on the interpretation of the NMR spectra[1].

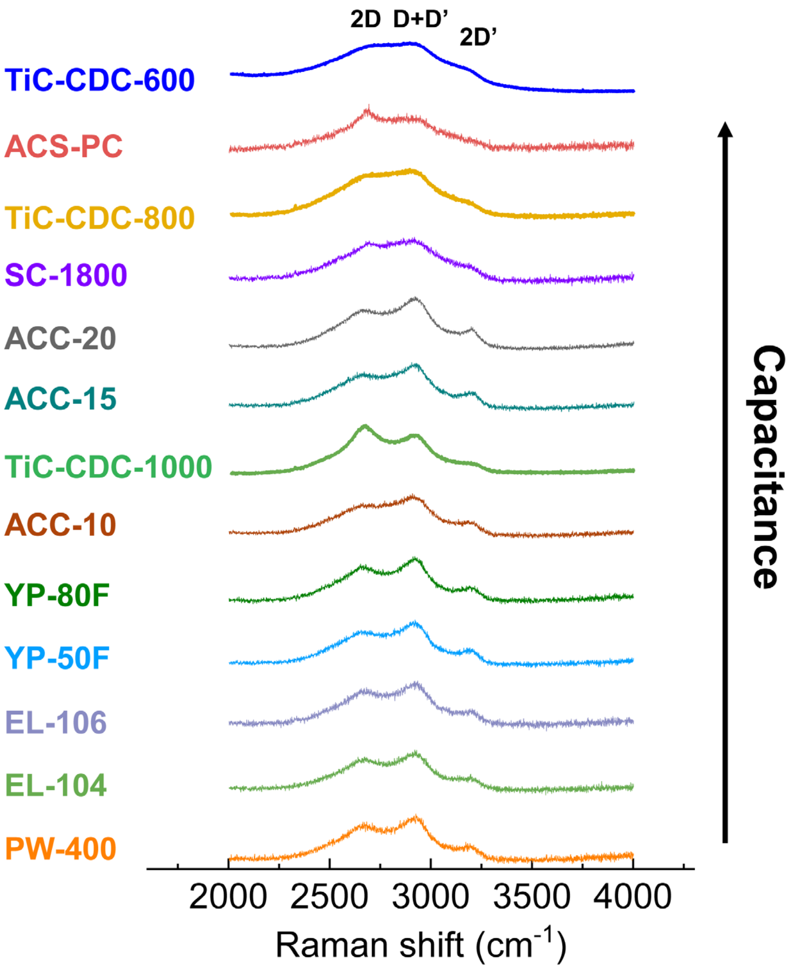

Figure S2. Raman spectra (532 nm) of ten commercial nanoporous carbons and three TiC-CDCs (532 nm) from previous literature at high Raman shifts, plotted in order of their increasing capacitance.[4, 5]

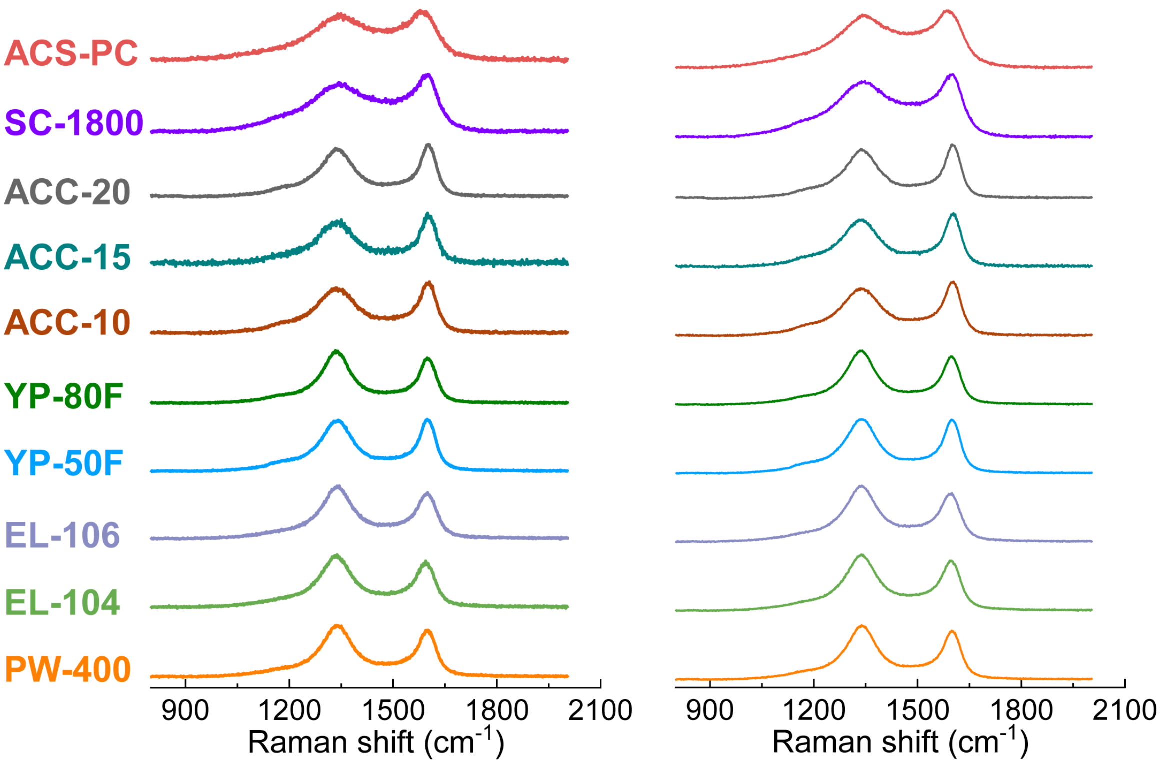

Figure S3. Comparison between a selected Raman spectrum (532 nm) for each sample (left) and the averaged Raman spectra from three to four different spots (right) of ten commercial nanoporous carbons. No significant differences are observed.

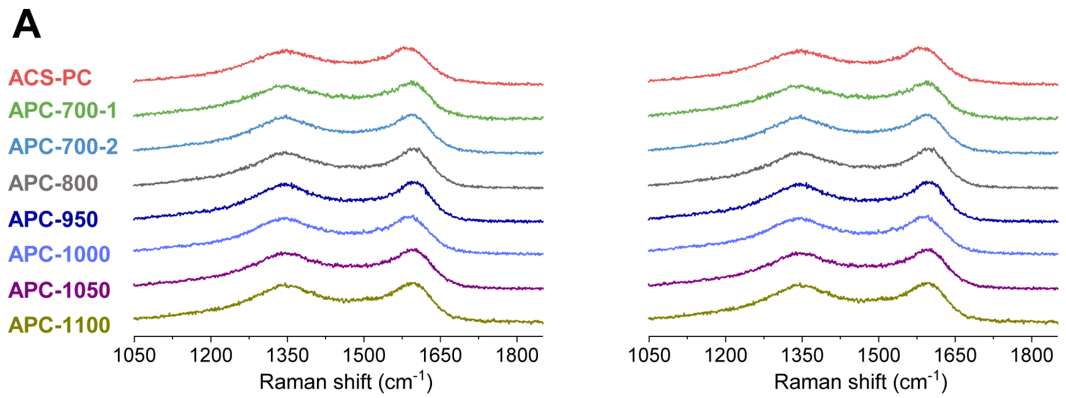

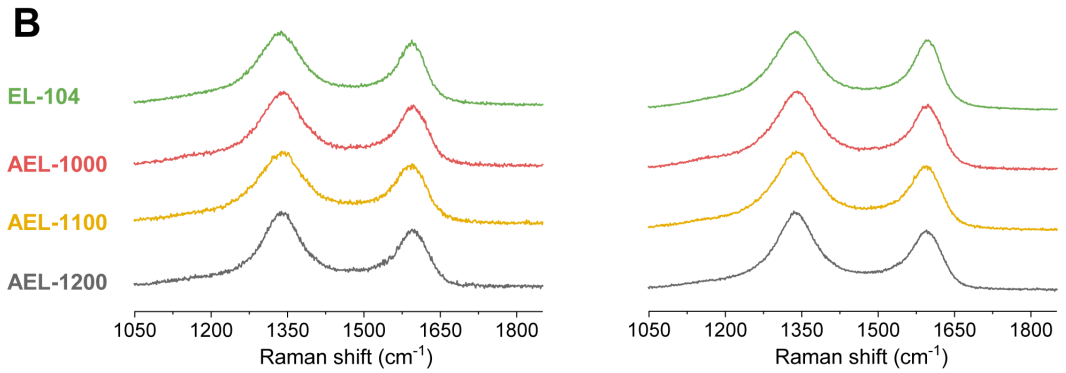

Figure S4. (A) Comparison between a selected Raman spectrum (532 nm) for each sample (left) and the averaged Raman spectra from different spots (right) of ACS-PC and thermally annealed ACS-PC. (B) Comparison between a selected Raman spectrum (532 nm) for each sample (left) and the averaged Raman spectra from different spots (right) of EL-104 and thermally annealed EL-104. No significant differences are observed.

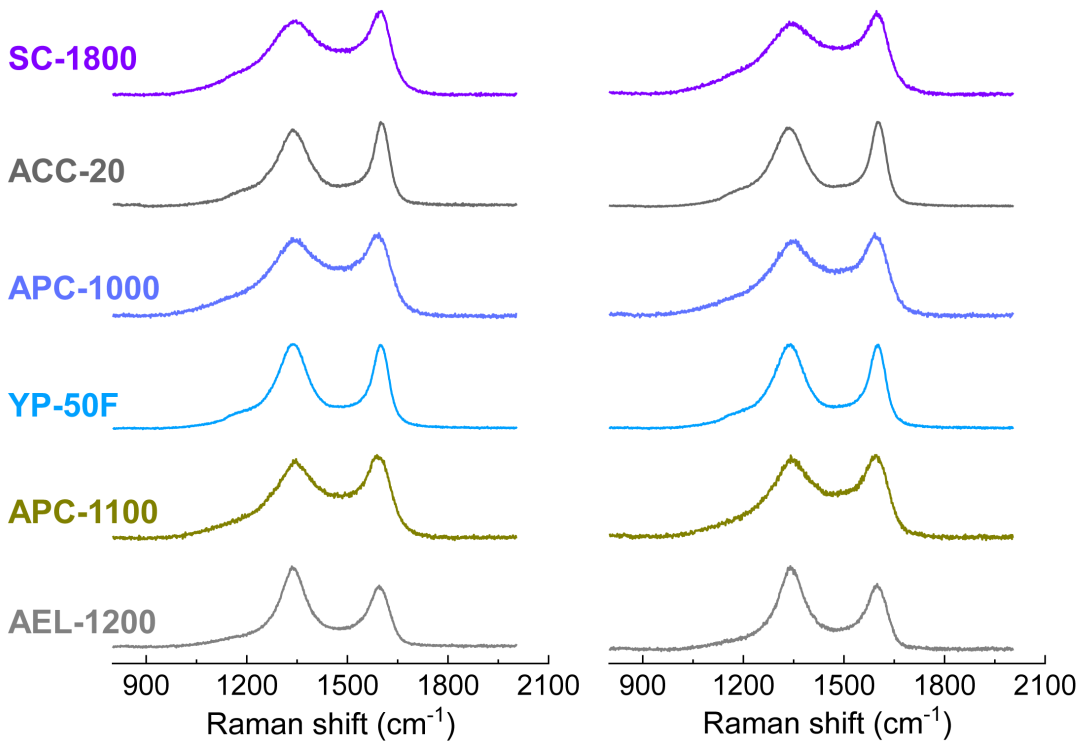

Figure S5. Comparison between Raman spectra (532 nm) acquired with a spot size diameter of approximately 400 to 500 nm (left) and spectra obtained with a larger spot size (×20 objective lens) (right) for six selected samples with varying degrees of structural order. No significant differences are observed.

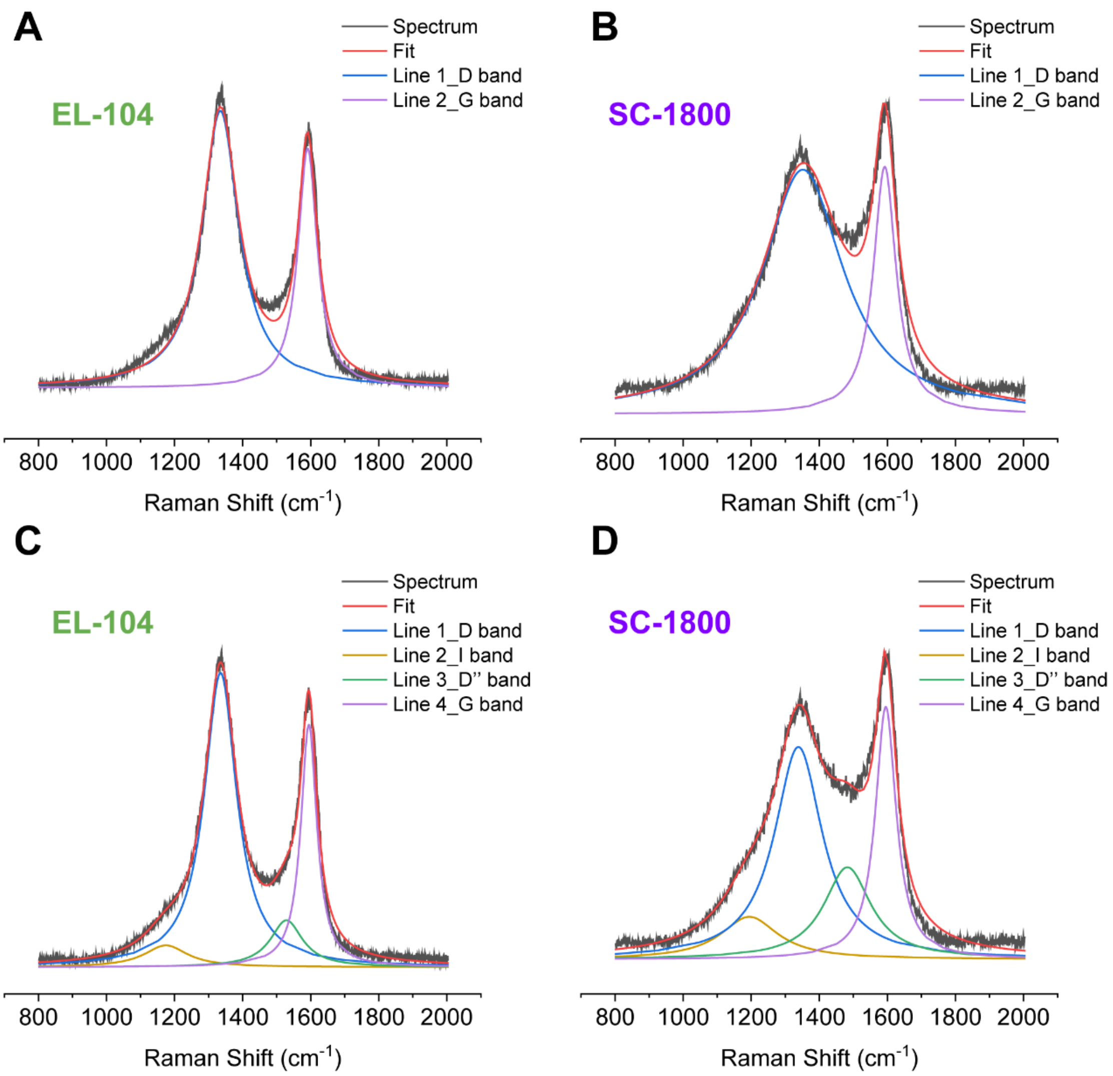

Figure S6. Comparison between 2-peak and 4-peak fitting models for nanoporous carbons with different degree of disorder. (A) 2-peak deconvolution of Raman spectrum of EL-104 (a more ordered carbon). (B) 2-peak deconvolution of Raman spectrum of SC-1800 (a more disordered carbon). (C) 4-peak deconvolution of Raman spectrum of EL-104 (a more ordered carbon). (D) 4-peak deconvolution of Raman spectrum of SC-1800 (a more disordered carbon). The application of the 4-peak analysis provides a more accurate fit for carbons with varying degrees of disorder.

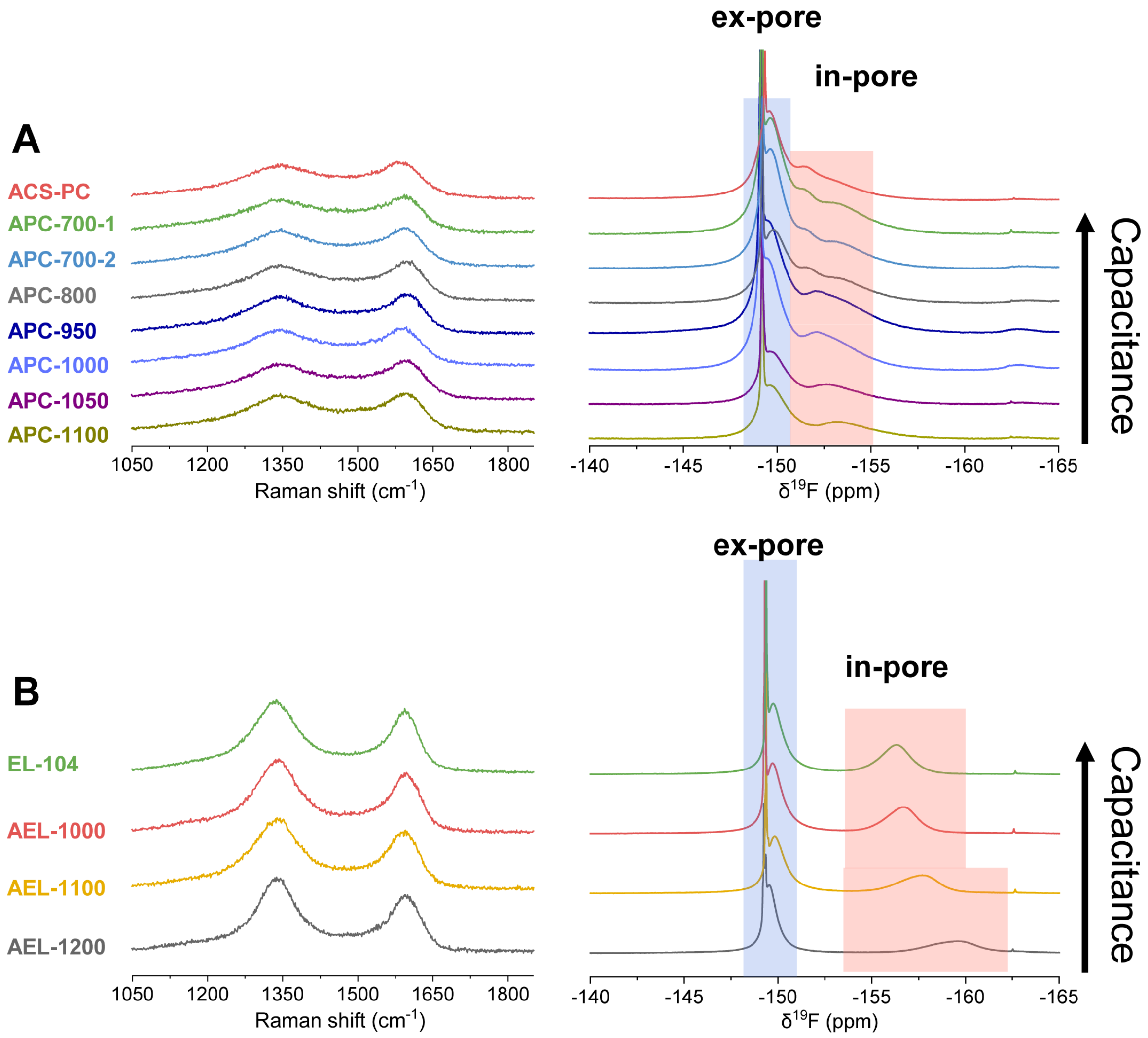

Figure S7. Comparison between Raman spectra (532 nm) of thermally annealed nanoporous carbons and ^{19}F MAS NMR spectra (9.4 T, 5 kHz MAS) of thermally annealed nanoporous carbons saturated with 1 M NEt_4BF_4 (ACN). (A) Comparison between Raman spectra (532 nm) (left) of thermally annealed ACS-PC and ^{19}F MAS NMR spectra (9.4 T, 5 kHz MAS) (right) of thermally annealed ACS-PC saturated with 1 M NEt_4BF_4 (ACN). (B) Comparison between Raman spectra (532 nm) (left) of thermally annealed EL-104 and ^{19}F MAS NMR spectra (9.4 T, 5 kHz MAS) (right) of thermally annealed EL-104 saturated with 1 M NEt_4BF_4 (ACN). For more details on the interpretation of the NMR spectra, see our previous work.[1]

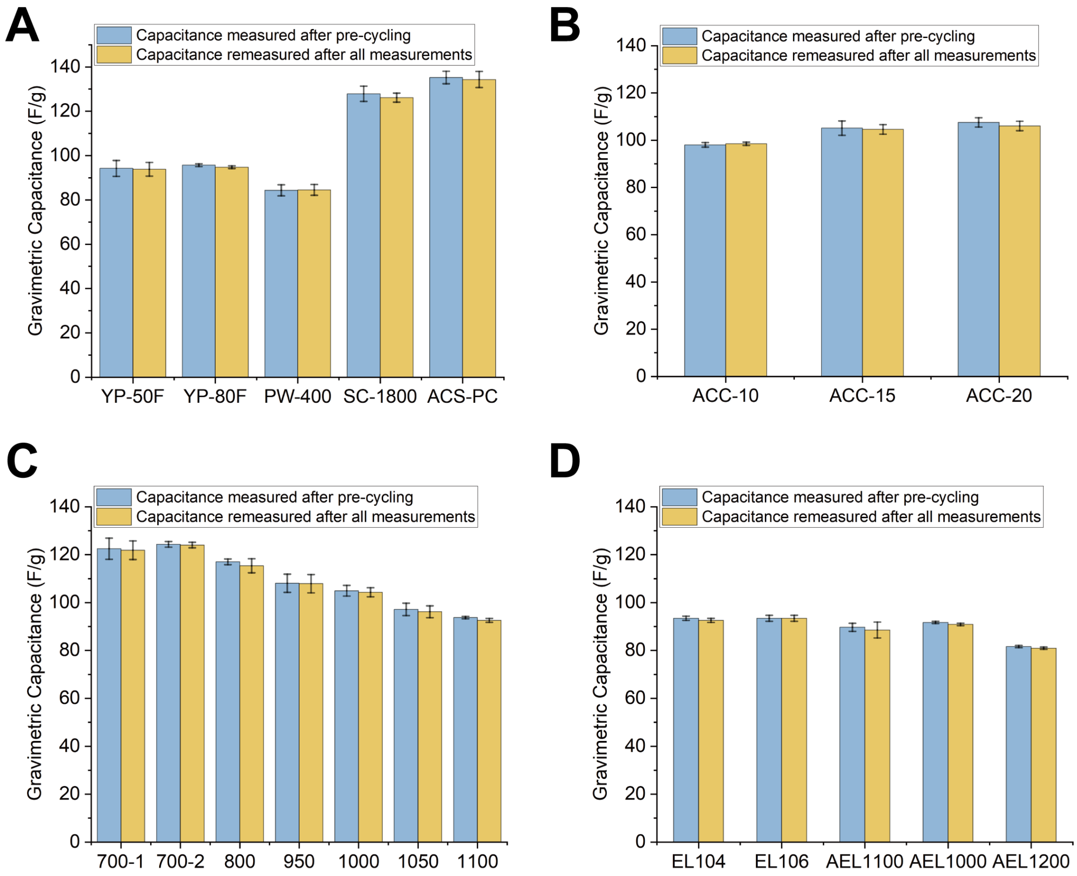

Fig. S8. Gravimetric Capacitance at 0.05 A/g measured immediately after pre-cycling and after all other electrochemical measurements. (A) Gravimetric capacitances of YP-50F, YP-80F, PW-400, SC-1800 and ACS-PC at 0.05 A/g in 1 M NEt_4BF_4 (ACN) after pre-cycling and after all other electrochemical measurements. (B) Gravimetric capacitances of ACC-10, ACC-15 and ACC-20 at 0.05 A/g in 1 M NEt_4BF_4 (ACN) after pre-cycling and after all other electrochemical measurements. (C) Gravimetric capacitances of annealed ACS-PC at 0.05 A/g in 1 M NEt_4BF_4 (ACN) after pre-cycling and after all other electrochemical measurements (D) Gravimetric capacitances of EL-104, EL-106 and annealed EL-104 at 0.05 A/g in 1 M NEt_4BF_4 (ACN) after pre-cycling and after all other electrochemical measurements. No obvious increase in capacitance is observed, indicating sufficient cell pre-cycling was carried out.

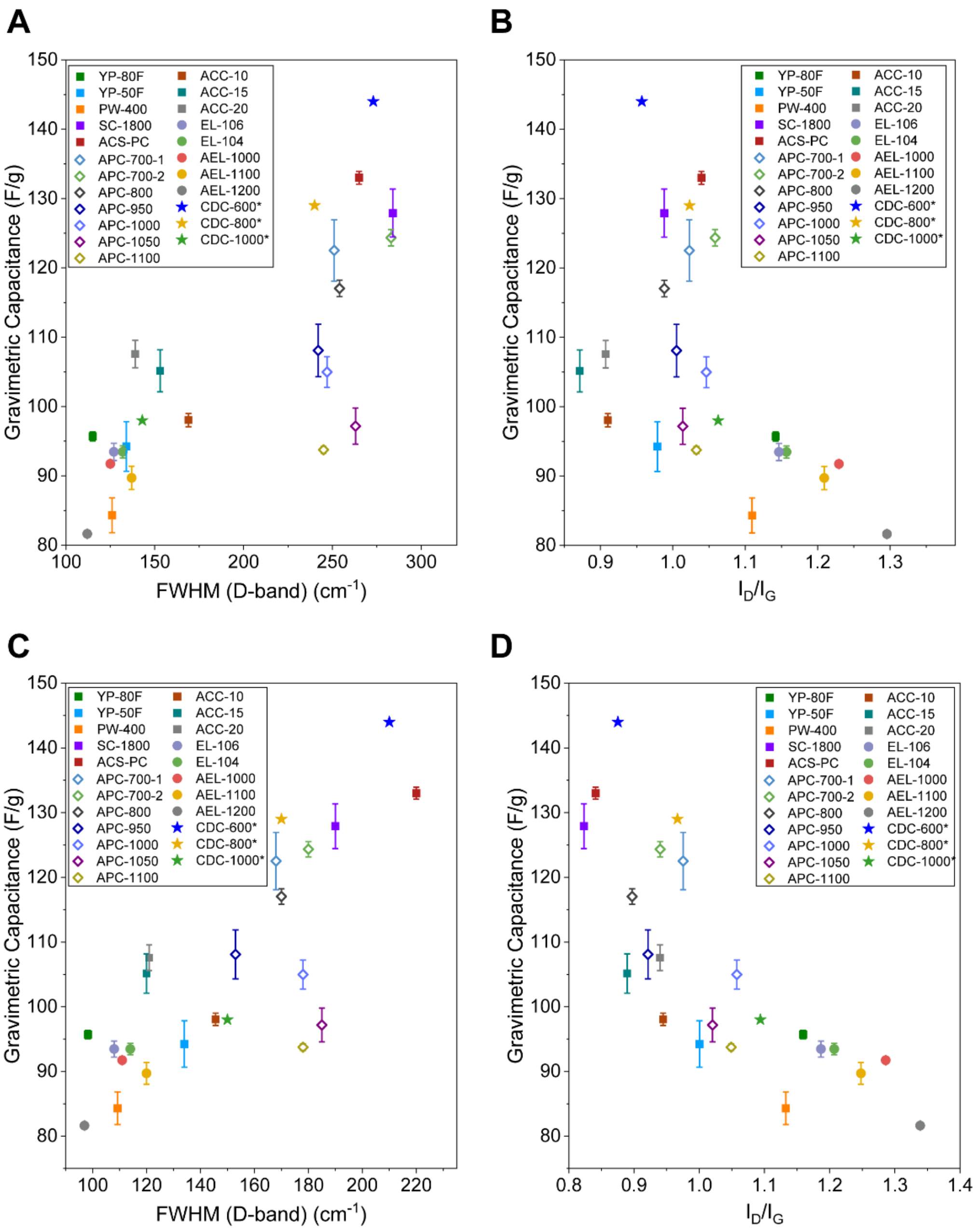

Figure S9. Relationship between gravimetric capacitance and parameters derived from Raman spectra using other fitting methods. (A) Relationship between gravimetric capacitance and D band FWHM of the studied carbons using a 2-peak model deconvolution. (B) Relationship between gravimetric capacitance and I_D/I_G intensity ratio of the studied carbons using a 2-peak model deconvolution. (C) Relationship between gravimetric capacitance and D band FWHM of the studied carbons using a 4-peak model deconvolution with constraints. (D) Relationship between gravimetric capacitance and I_D/I_G intensity ratio of the studied carbons using a 4-peak model deconvolution with constraints. Generally, the trends are consistent with those in Figure 2, regardless of the fitting models – however, as discussed above, the 4 peak models provide better fits of the Raman data. Nanoporous carbons with larger D band FWHM and smaller I_D/I_G values show higher capacitance.

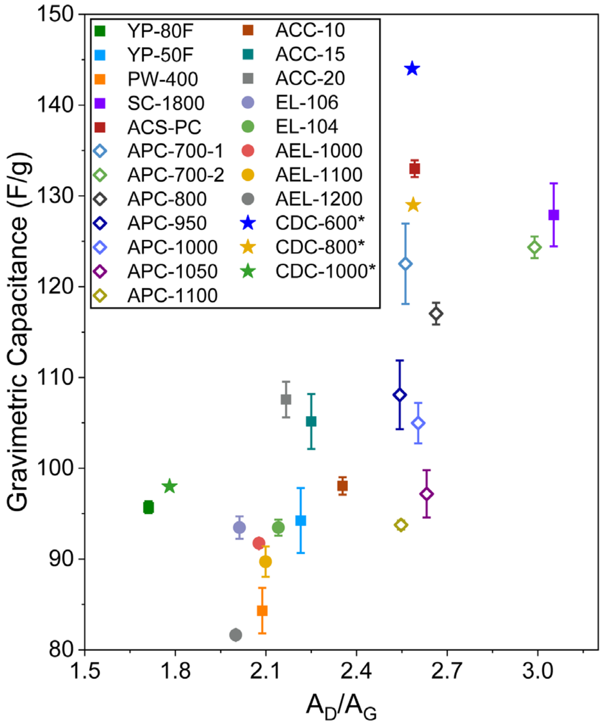

Figure S10. Relationship between gravimetric capacitance and integrated area ratio of D band to G band (A_D/A_G) of the studied carbons using a 2-peak model deconvolution. In general, the capacitance is proportional to A_D/A_G for the studied carbons. However, the trend is weaker in part because the intensity (strictly, peak height) and FWHM both track disorder, but with smaller I_D/I_G values and larger D band FWHM for the more disordered carbons, and vice versa for the more ordered carbons.

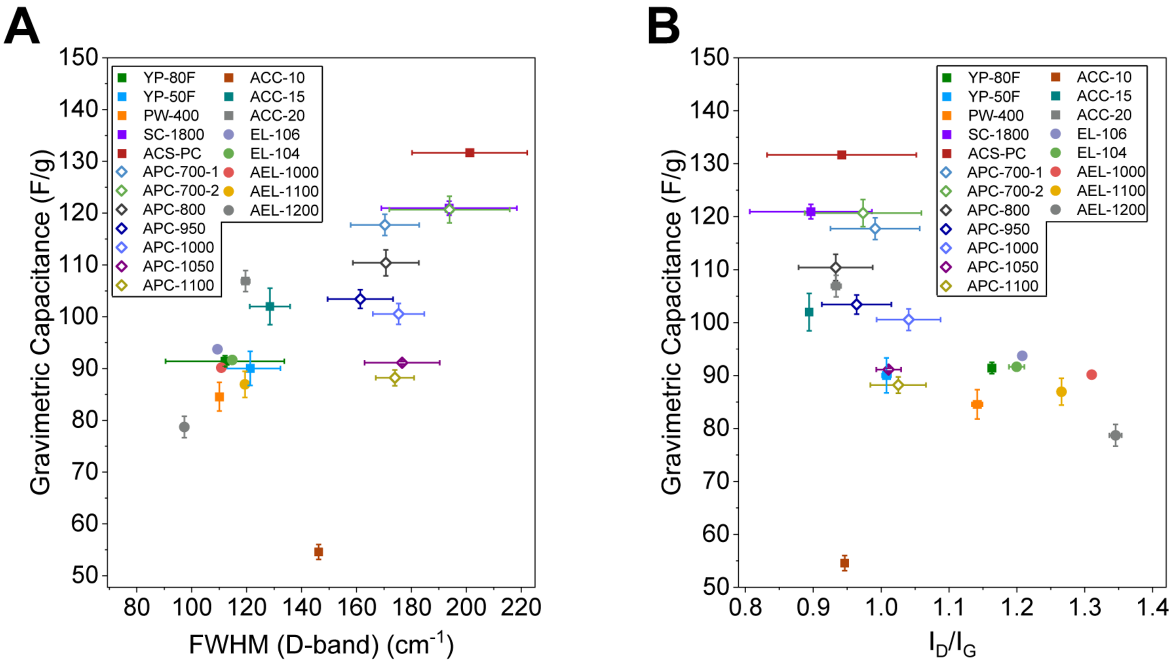

Figure S11. Relationship between gravimetric capacitance at 1 A/g and parameters derived from Raman spectra. (A) Relationship between gravimetric capacitance and D band FWHM of the studied carbons. (B) Relationship between gravimetric capacitance and I_D/I_G intensity ratio (i.e. peak height intensity ratio) of the studied carbons. Gravimetric capacitances of commercial nanoporous carbons and their thermally annealed counterparts were measured in two-electrode symmetric coin cells at 1 A/g in 1 M NEt_4BF_4 (acetonitrile, ACN). Overall, the correlation still remains, with an outlier observed (ACC-10) at high current density due to limited porosity. This suggests that kinetic performance is influenced by structural factors beyond the structural order degree, which is out of the scope of Raman spectroscopy.

Table S1. Table of gravimetric capacitance of the studied carbons. Gravimetric capacitances of commercial nanoporous carbons were measured in two-electrode symmetric coin cells at 0.05 A/g in 1 M NEt_4BF_4 (acetonitrile, ACN). Capacitances of TiC-CDCs were extracted from previous literature,[5] measured in two-electrode cell at 5 mA/cm² in 1.5 M NEt_4BF_4 (ACN).

Carbon Name	Gravimetric Capacitance (F/g)
YP-50F	94.3±3.6
YP-80F	95.7±0.7
PW-400	84.3±2.5
SC-1800	127.9±3.4
ACS-PC	133.0±1.0
EL-104	93.5±0.9
EL-106	93.5±1.2
ACC-10	98.1±1.0
ACC-15	105.1±3.0
ACC-20	107.6±2.0
APC-700-1	122.5±4.4
APC-700-2	124.3±1.2
APC-800	117.0±1.2
APC-950	108.1±3.8
APC-1000	105.0±2.2
APC-1050	97.2±2.6
APC-1100	93.8±0.5
AEL-1000	91.7±0.5
AEL-1100	89.7±1.7
AEL-1200	81.6±0.5
TiC-CDC-600	144
TiC-CDC-800	129
TiC-CDC-1000	98

References

(1) Liu, X. Y.; Lyu, D. X.; Merlet, C.; Leesmith, M.; Hua, X.; Xu, Z.; Grey, C. P.; Forse, A. C. Structural disorder determines capacitance in nanoporous carbons. *Science* **2024**, *384* (6693), 321-325. DOI: 10.1126/science.adn6242.

(2) Merlet, C.; Forse, A. C.; Griffin, J. M.; Frenkel, D.; Grey, C. P. Lattice simulation method to model diffusion and NMR spectra in porous materials. *J Chem Phys* **2015**, *142* (9). DOI: 10.1063/1.4913368.

(3) Forse, A. C.; Griffin, J. M.; Presser, V.; Gogotsi, Y.; Grey, C. P. Ring Current Effects: Factors Affecting the NMR Chemical Shift of Molecules Adsorbed on Porous Carbons. *J Phys Chem C* **2014**, *118* (14), 7508-7514. DOI: 10.1021/jp502387x.

(4) Forse, A. C.; Merlet, C.; Allan, P. K.; Humphreys, E. K.; Griffin, J. M.; Aslan, M.; Zeiger, M.; Presser, V.; Gogotsi, Y.; Grey, C. P. New Insights into the Structure of Nanoporous Carbons from NMR, Raman, and Pair Distribution Function Analysis. *Chem. Mater.* **2015**, *27* (19), 6848-6857. DOI: 10.1021/acs.chemmater.5b03216.

(5) Chmiola, J.; Yushin, G.; Gogotsi, Y.; Portet, C.; Simon, P.; Taberna, P. L. Anomalous increase in carbon capacitance at pore sizes less than 1 nanometer. *Science* **2006**, *313* (5794), 1760-1763. DOI: 10.1126/science.1132195.
